# Engineering of *Trichoderma reesei* for enhanced degradation of lignocellulosic biomass by truncation of the cellulase activator ACE3

**DOI:** 10.1186/s13068-020-01701-3

**Published:** 2020-04-01

**Authors:** Yumeng Chen, Chuan Wu, Xingjia Fan, Xinqing Zhao, Xihua Zhao, Tao Shen, Dongzhi Wei, Wei Wang

**Affiliations:** 1 State Key Lab of Bioreactor Engineering, East China University of Science and Technology, P.O.B. 311, 130 Meilong Road, Shanghai, 200237 China; 2 State Key Laboratory of Microbial Metabolism, Joint International Research Laboratory of Metabolic & Developmental Sciences, School of Life Sciences and Biotechnology, Shanghai Jiao Tong University, Shanghai, 200240 China; 3 College of Life Science, Jiangxi Normal University, Nanchang, 330022 China; 4 Sunson Industry Group Co, Ltd,, Beijing, China

**Keywords:** *Trichoderma reesei*, Genetic engineering, Truncated ACE3, Cellulase production, Lignocellulosic biomass

## Abstract

**Background:**

The filamentous fungus *Trichoderma reesei* is a major workhorse employed to produce cellulase, which hydrolyzes lignocellulosic biomass for the production of cellulosic ethanol and bio-based products. However, the economic efficiency of biorefineries is still low.

**Results:**

In this study, the truncation of cellulase activator ACE3 was identified and characterized in *T. reesei* classical mutant NG14 and its direct descendants for the first time. We demonstrated that the truncated ACE3 is the crucial cause of cellulase hyper-production in *T. reesei* NG14 branch. Replacing the native ACE3 with truncated ACE3 in other *T. reesei* strains remarkably improves cellulase production. By truncating ACE3, we engineered a *T. reesei* mutant, PC-3-7-A723, capable of producing more cellulase than other strains. In a 30-L fermenter, fed-batch fermentation with PC-3-7-A723 drastically increased the maximum cellulase titer (FPase) to 102.63 IU/mL at 240 h, which constitutes a 20–30% improvement to that of the parental strain PC-3-7.

**Conclusions:**

This work characterized the function of truncated ACE3 and demonstrated that analysis of classical mutants allows rational engineering of mutant strains with improved cellulase production necessary to process lignocellulosic biomass. Our rational engineering strategy might be useful for enhancing the production of other bio-based products.

## Background

The main product from lignocellulose degradation is glucose that is then converted into value-added products, such as ethanol [1–4], which have attracted increasing interest. Lignocellulosic biomass, which represents the most abundant sustainable resource, is a key player in the production of biofuels and bio-based chemicals in biorefineries [5]. One of the crucial steps of lignocellulosic biomass degradation is enzymatic hydrolysis, and filamentous fungi are the most successful microorganisms for producing lignocellulolytic enzymes [1]. Among these, the saprophytic filamentous fungus *Trichoderma reesei* (teleomorph *Hypocrea jecorina*) is a dominant workhorse employed for cellulase production in the biotechnology industry [6]. However, large-scale conversion of lignocellulose to products is suffering from the high cost of cellulase.

**Fig. 1:**
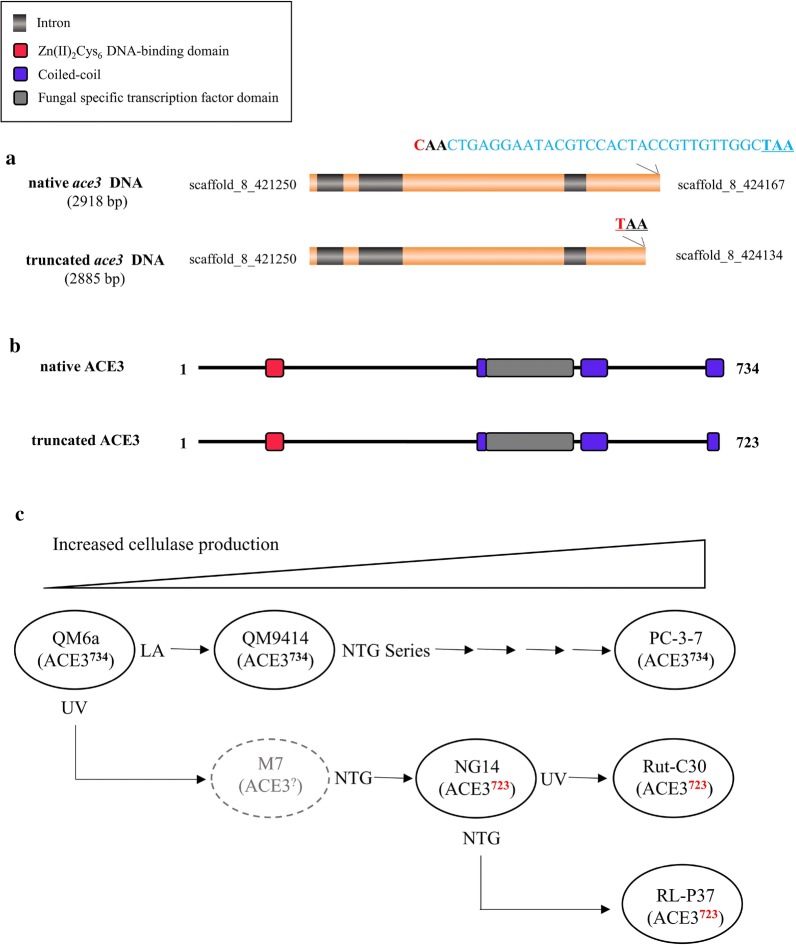
Native and truncated ACE3 sequences and genealogy of strains used in this study. **a** Native and truncated *ace3* DNA sequences. Red color represents the missense mutation loci in the sequence of *ace3*. Blue color represents the DNA sequence of the 11 truncated amino acids. The underlines indicate the stop codons. **b** Native type ACE3-734 and truncated type ACE3-723. Native ACE3 is composed of 734 amino acids, and truncated ACE3-723 is composed of 723 amino acids. **c** Genealogy of strains used in this study. Mutagens used appear next to strain names. The gray color used for the M7 strain indicates that the strain is no longer available and was not included in this study. The number 723 denoted in red represents the strain containing truncated ACE3-723. *LA* Linear accelerator; *UV* ultraviolet light; *NTG* N-nitroguanidine

*Trichoderma reesei* Rut-C30, one of the best-known hyper-producers of cellulase in the public domain, was obtained through three rounds of classical mutagenesis from the wild-type QM6a (Fig. 1c) [6, 7]. First, mutagenesis using UV (ultraviolet light) followed by screening for the ability to hydrolyze cellulose led to the isolation of the M7 strain (which is no longer available). Further mutagenesis using NTG (N-nitroguanidine) led to the isolation of the hyper-producer strain NG14 that exhibited two- to fivefold increased cellulase activity compared to the QM6a parental strain [8]. Finally, the *T. reesei strain* Rut-C30 was obtained using another round of UV mutagenesis, followed by screening for improved cellulase activity compared to the NG14 parental strain. In addition, an RL-P37 strain was also selected from NG14 (Fig. 1c) [9]. Meanwhile, a moderately overproducing strain of cellulase (QM9414) was derived from QM6a by irradiation using a linear accelerator [7]. *T. reesei* strain PC-3-7 [10], derived from QM9414 through several rounds of mutagenesis, is a cellulase hyper-producing mutant that exhibits twice as much cellulase activity as QM9414 [11]. This has led to the identification of a large number of mutagenic events that contribute to the improvements of cellulase in the QM9414 and NG14 branch [5, 12–14]. However, most of the mutation sites are found in non-coding regions of the genome during classical mutagenesis, which makes it hard to understand their role in cellulase hyper-production. To date, the genomes of some strains have been sequenced [8, 15], thus making the organism open to targeted improvement by genetic engineering.

Genetic engineering is a feasible strategy to increase cellulase production, for example by manipulating transcription factors (TFs) to regulate gene expression [1, 16]. The *bgl2* gene encoding intracellular β-glucosidase II (BGLII/Cel1a) is mutated in PC-3-7; this mutation reduces the cellobiose metabolic rate, resulting in an indirect release from carbon catabolite repression of cellulase expression by glucose, which enhances cellulase expression [13]. The engineering of TFs involved in transcription of cellulase genes has been developed to enhance the production of cellulase in filamentous fungi [5, 17]. An artificial TF containing the carbon catabolite repression factor CRE1-binding domain linked to the cellulase essential TF XYR1 was designed, which results in constitutive cellulase production using glucose as the sole carbon source [18]. Derntl et al. [19] introduced fusion TFs consisting of the DNA-binding domain of XYR1 and the transactivation domain of YPR2 (a TF of the sorbicillinoid biosynthesis gene cluster), which yielded a highly transactivating TF that induced xylanases and cellulases near-independently from a carbon source. Wang et al. [20] designed four novel fusion TFs to release or attenuate carbon catabolite repression in cellulase transcription, which led to significant improvement of cellulase gene expression. Overall, genetic engineering could aim to optimize regulatory processes within cells to increase cellulase production.

In general, the induction of cellulase gene expression is triggered by specific inducer molecules. After recognition of the inducer, TFs are activated and bound to cellulase-encoding genes [1, 21]. Several particular mutations located in these TFs cause cellulase hyper-production in classical mutants. The CRE1 mutations in *T. reesei* Rut-C30 and PC-3-7, which allow the transcription of cellulase genes in glucose-containing media, resulted in higher cellulase and hemicellulase activities [7, 22, 23]. Nitta et al. [24] indicated that the mutation of BGLR (a TF that regulates β-glucosidase gene expression) in PC-3-7 elevated cellulase production during growth on cellobiose.

In 2014, another cellulase essential TF, ACE3, was identified for the first time from the transcriptional profiling data of *T. reesei* cultures grown in the presence of different lignocellulose-derived cellulase inducers [25]. Deletion of the ACE3 encoding gene *ace3* is fatal for cellulase production and for the transcription of several cellulase genes studied, which proved ACE3 is an essential TF for cellulase [25]. We have identified the correct sequence of the native ACE3 (ACE3-734, encoding 734 amino acids) from *T. reesei* QM6a [26] and then elucidated its essential role in cellulase production, by observing its interaction with another essential cellulase TF, XYR1 [26].

In this study, we analyzed the genomes of the classical *T. reesei* mutants to find the mutations responsible for the remarkable improvement of cellulase production. We engineered a *T. reesei* hyper-cellulolytic mutant by integration of the crucial mutations from two *T. reesei* classical mutagenesis branches. Furthermore, higher FPase titer and cellulase productivity were achieved using a low-cost sugar mixture in a 30-L fermenter by fed-batch fermentation. Our study provides a rational engineering strategy to improve cellulase production, which could be further elaborated to feasibly improve cellulosic ethanol production and might benefit efficient bioconversion of other bio-based products.

## Results

### Identification of the mutations in TFs from *T. reesei* classical mutants

Engineering of TFs has been the major strategy for increasing the production of lignocellulolytic enzymes in filamentous fungi. There are many mutagenic events occurring in TFs that contribute to the improvement of cellulases in different *T. reesei* classical hyper-producing mutants. We scanned the never-studied mutations located in TFs for cellulase production from the genome data of classical mutagenic strains to determine whether such mutations can contribute to the improvements of cellulase or not.

The genomes of the two *T. reesei* strains QM6a (wild type) and Rut-C30 (classical hyper-producing mutant) have been sequenced and published before [8, 15]. By comparing the two genome data, we discovered a point mutation in the Rut-C30 *ace3* gene, which results in a missense mutation (C to T, 2883 base pairs downstream of the *ace3* translational initiation), then in a premature termination of translation, and in a truncated ACE3 (Fig. 1a). The final *T. reesei* Rut-C30 product, ACE3-723, is 11 amino acids shorter at the C-terminus (Fig. 1a and Additional file 1: Figure S1). As shown in Fig. 1b, the open reading frame (ORF) of the native *ace3* encodes a protein that is composed of 734 amino acids, while the ORF of truncated *ace3* encodes a protein that is composed of 723 amino acids.

To further understand the missense mutation of *ace3* in *T. reesei*, we combined the results of sequencing data and diagnostic PCR to characterize the sequences in other classical mutants that were derived from the *T. reesei* wild-type QM6a, such as QM9414, PC-3-7, NG14, and RL-P37. Consequently, we discovered that the *T. reesei* strains NG14 and RL-P37 contained the same missense mutation in the *ace3* sequence, and ACE3 was truncated to ACE3-723 in the same manner as in Rut-C30. However, in *T. reesei* QM9414 and PC-3-7, the ACE3 was found to be the same native ACE3-734 as that in wild-type QM6a (Fig. 1c). These results suggest that the truncation of *ace3* in *T. reesei* Rut-C30 and RL-P37 was inherited from their parental strain, NG14. However, the *T. reesei* QM9414 branch carries the native ACE3-734 without any mutation, thus indicating that the sequences of ACE3 are different between the NG14 and QM9414 descendants. These results imply that the truncation of ACE3 is specific to the NG14 branch that was developed by Peterson et al. [7] and requires further investigation to better understand its biological function and significance.

To date, there are no reports on the impact of ACE3 truncation on the function of this protein. Our study is the first to report the truncated mutation of *ace3* in a variety of *T. reesei* strains and the impact of this truncation on the biological function of ACE3 in detail.

### The truncated ACE3-723 is the crucial cause of cellulase hyper-production in *T. reesei* NG14 branch

**Fig. 2:**
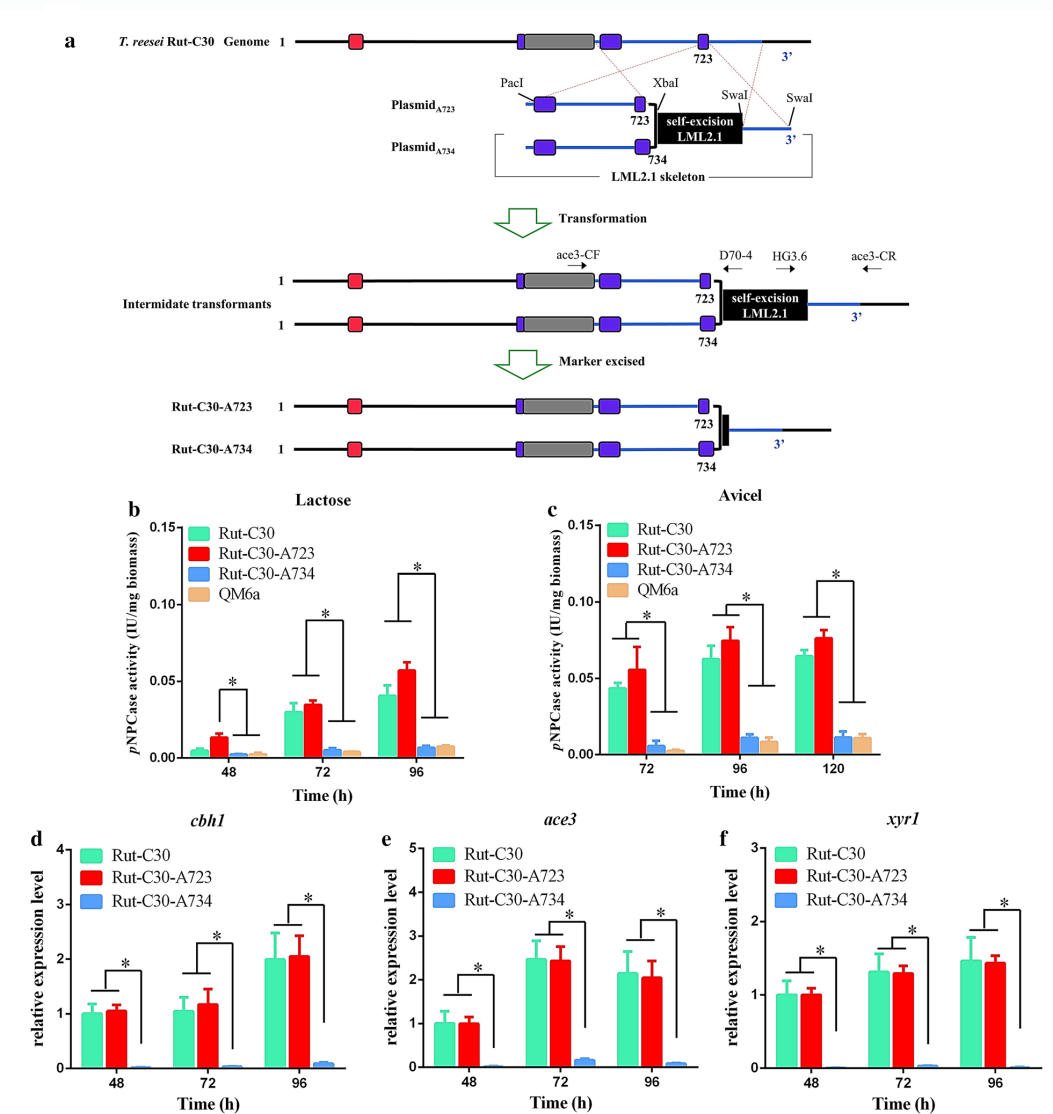
Construction of transformants and effects of the truncated type ACE3-723 versus the native type ACE3-734 on cellulase production of *T. reesei* Rut-C30. **a** Schematic representation of A723 and A734 transformants. LML 2.1 is the erasable hygromycin selection marker in *T. reesei*. A734 transformants carry the native ACE3-734 as the test strains. A723 transformants bear ACE3-723 as controls. The black square denotes the *lox*P site left at the C-terminus of ACE3 after the marker was excised. The primers ace3-CF and D70-4 and HG3.6 and ace3-CR were used to verify the genotype of ACE3. **b**, **c** Effects of the truncated type ACE3-723 versus the native type ACE3-734 on cellulase production of *T. reesei*. The *p*NPCase activity of *T. reesei* Rut-C30 and transformants was examined after culture in liquid 2× Mandels’ medium with 2% lactose (**b**) or 1% Avicel (**c**). **d**–**f** Transcription of genes encoding the major cellulase (*cbh1*) and essential transcription factors for cellulase (*ace3* and *xyr1*) was evaluated. Three independent experiments with three biological replicates each were performed. The *sar1* gene was used as the internal control for normalization. Values are the mean ± SD of the results from three independent experiments. Asterisks indicate a significant difference (*p < 0.05, Student’s *t* test)

The identification of a truncated ACE3-723 in Rut-C30 led us to investigate whether it is involved in its cellulase hyper-production. To directly test this hypothesis, we replaced the truncated *ace3*-*723* sequence in the *T. reesei* Rut-C30 with the native *ace3*-*734* sequence from *T. reesei* QM6a using in situ homologous recombination, thus creating two transformants for the *T. reesei* Rut-C30 strain (Fig. 2a). Rut-C30-A734 transformants carried the native ACE3-734, as the test strains did, while the Rut-C30-A723 transformants carried the truncated type ACE3-723 found in Rut-C30 and were used as a positive control. All transformants were confirmed to have the correctly replaced gene, with a single-copy DNA integration only (Additional file 2; Additional file 3: Figure S2).

Next, we cultured all the strains on liquid 2× Mandels’ medium with either 2% lactose or 1% Avicel as the sole carbon source. The titer of *p*NPCase activity (representing cellulase activity in our study) was analyzed. As shown in Fig. 2b, c, there was no significant difference in cellulase production from the positive control (Rut-C30-A723 transformants) compared to that of parental strain Rut-C30, which can be explained by them having the same *ace3*-*723* genotype. However, the titers of *p*NPCase activity were significantly reduced in the test Rut-C30-A734 transformants compared to those in the parental strain (Rut-C30) and the positive control (Rut-C30-A723 transformants). Interestingly, we found that the titer of *p*NPCase activity in the test Rut-C30-A734 transformants was dramatically decreased to that of the original level observed in wild-type QM6a (Fig. 2b, c). This result suggests that the complementing native *ace3*-*734* completely blocked their hyper-production trait, which was acquired by several rounds of classical mutagenesis in a course of 30 years.

In addition, the transcriptional levels of *cbh1* (encoding the major cellulase, cellobiohydrolase I in *T. reesei*), *xyr1* (major transcriptional regulator of cellulases), and *ace3* (essential cellulase activator) were also analyzed using RT-qPCR (RNA extraction and real-time reverse transcription polymerase chain reaction) in the Rut-C30 strain, in the positive control Rut-C30-A723 transformants, and in the test Rut-C30-A734 transformants grown on 1% Avicel following 48, 72, and 96 h of induction. The transcription levels of *cbh1*, *xyr1*, and *ace3* in Rut-C30-A723 and Rut-C30 are similar (Fig. 2d, e), which can be explained by their common *ace3*-*723* genotype. However, the transcription levels of *cbh1*, *xyr1*, and *ace3* in the test Rut-C30-A734 were about 40-, 120-, and 40-fold lower than those in the ACE3-723 genotype strains, respectively (Fig. 2d, e). The RT-qPCR analyses of *cbh1* (Fig. 2d, e) agreed with the cellulase activity analyses (Fig. 2b, c). The marked down-regulated transcription levels of *xyr1* and *ace3* in Rut-C30-A734 (Fig. 2d, e) could explain its significantly scarcer cellulase production (Fig. 2b, c). Hence, results strongly support the thesis that the truncated type ACE3-723 is the crucial cause for cellulase hyper-production in Rut-C30.

To further investigate whether the truncated ACE3-723 in *T. reesei* NG14 and RL-P37 is also involved in the hyper-production of cellulase like in the Rut-C30 strain, we replaced the truncated *ace3*-*723* sequence in the *T. reesei* NG14 and RL-P37 with the native *ace3*-*734* sequence from *T. reesei* QM6a using in situ homologous recombination; this resulted in two transformants, NG14-A734 and RL-P37-A734, designed to carry the native ACE3-734 and be the test strains for NG14 and RL-P37, respectively. The NG14-A723 and RL-P37-A723 transformants possessed the same ACE3-723 proteins as their parental strains NG14 and RL-P37, which were used as their positive controls, respectively.

**Fig. 3:**
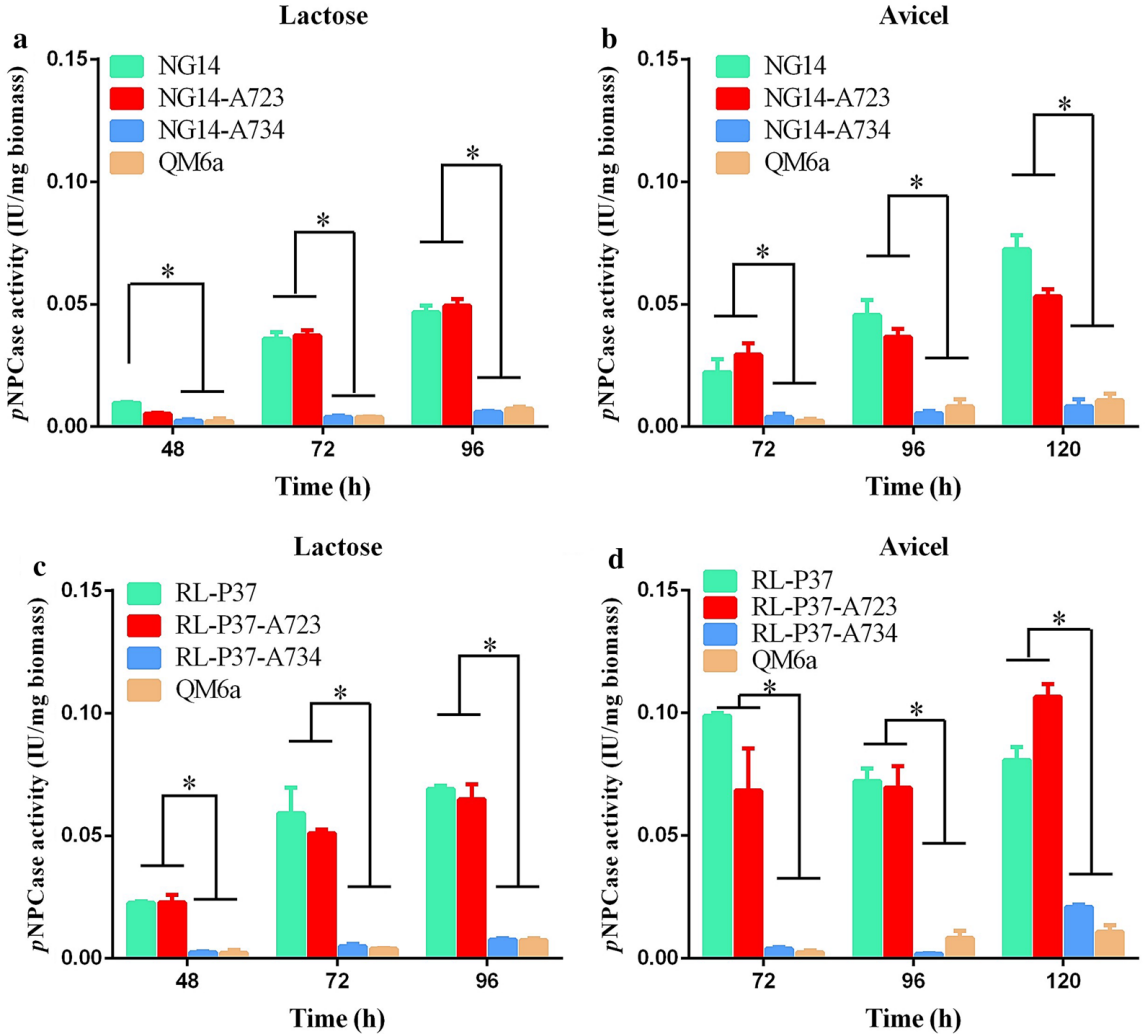
Effects of the truncated type ACE3-723 versus the native type ACE3-734 on cellulase production of *T. reesei* NG14 and RL-P37. The *p*NPCase activity of *T. reesei* NG14 (**a**, **b**) and RL-P37 (**c**, **d**) was examined after culture in liquid 2× Mandels’ medium with 2% lactose (**a**, **c**) or 1% Avicel (**b**, **d**). Values are the mean ± SD of results from three independent experiments. Asterisks indicate a significant difference (*p < 0.05, Student’s *t* test)

The titer of *p*NPCase activity was analyzed. As shown in Fig. 3, there was no significant difference in cellulase production in the positive controls (NG14-A723 and RL-P37-A723 transformants) compared to parental strains (NG14 and RL-P37, respectively), which can be explained by the common genotype held by each pair of parental and positive control strain. However, the titers of *p*NPCase activity were significantly reduced in the test NG14-A734 and RL-P37-A734 transformants compared to those in parental stains (NG14 and RL-P37, respectively) and positive controls (NG14-A723 and RL-P37-A723 transformants, respectively). Similar to Rut-C30-A734 transformants, the titer of *p*NPCase activity in NG14-A734 and RL-P37-A734 transformants was dramatically decreased to that of the original level observed in wild-type QM6a (Fig. 3). The transcriptional levels of *cbh1*, *xyr1*, and *ace3* were also analyzed using RT-qPCR in the NG14 group (NG14, NG14-A723, and NG14-A734) and RL-P37 group (RL-P37, RL-P37-A723, and RL-P37-A734) (Additional file 4: Figure S3), and resulted similar to those of the Rut-C30 group (Rut-C30, Rut-C30-A723, and Rut-C30-A734). This result is completely consistent with that of Rut-C30, and indicates that the truncated type ACE3-723 is the crucial cause of the cellulase hyper-production in NG14 and its descendants Rut-C30 and RL-P37 (Fig. 1c). We may speculate that if 50 years ago the classic random mutagenesis had not caused this missense mutation in the *ace3* sequence, the NG14 would have been discarded as a low-yield cellulase-producing strain, and hence its descendants Rut-C30 and RL-P37 would not exist.

### Engineered PC-3-7-A723 produces more cellulase than other *T. reesei* strains

The results documented in Figs. 2 and 3 suggest that the truncation of 11 amino acids at the C-terminus of ACE3-734 is the crucial cause of the cellulase hyper-production in NG14 and its descendants Rut-C30 and RL-P37. We sought to determine whether rational genetic engineering could improve cellulase production by integration of this crucial mutation into the *T. reesei* strains QM6a, QM9414, and PC-3-7, which still contain native *ace3*-*734*. To study this, we replaced the native *ace3*-*734* sequence in *T. reesei* QM6a, QM9414, and PC-3-7 with a truncated *ace3*-*723* sequence from *T. reesei* Rut-C30 by in situ homologous recombination, engineering in two kinds of transformants for each strain (see Additional file 5: Figure S4A). The A723 transformants carried the truncated ACE3-723 as the test strains. The A734 transformants carried the native ACE3-734 sequence like the parental strains, which were used as the positive controls.

**Fig. 4:**
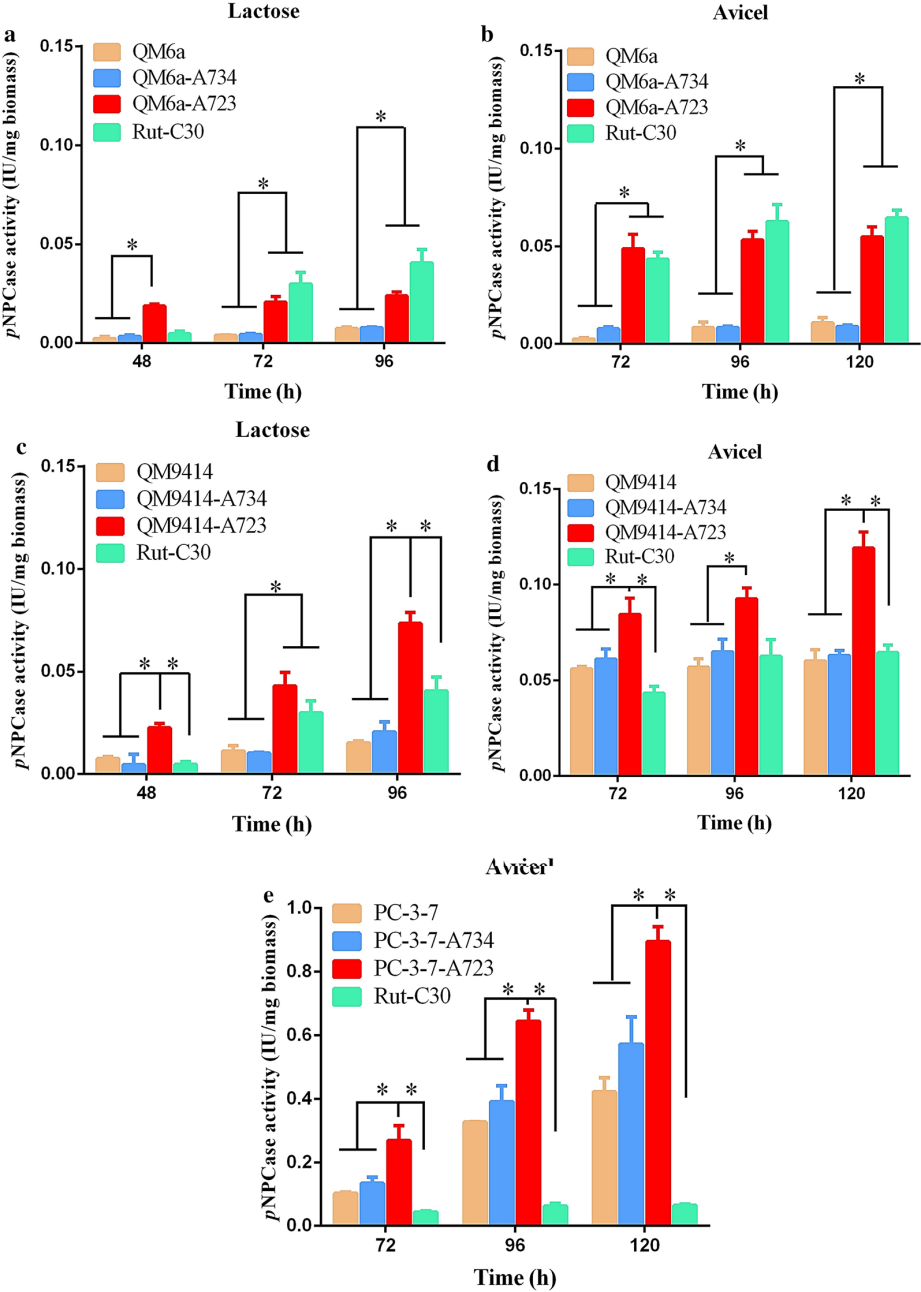
Effects of the truncated type ACE3-723 versus the native type ACE3-734 on cellulase production in *T. reesei* QM6a, QM9414, and PC-3-7. The *p*NPCase activity of *T. reesei* QM6a (**a**, **b**), QM9414 (**c**, **d**), and PC-3-7 (**e**) was examined after culture in liquid 2× Mandels’ medium with 2% lactose (**a**, **c**) or 1% Avicel (**b**, **d**, **e**). Values are the mean ± SD of the results from three independent experiments. Asterisks indicate a significant difference (*p < 0.05, Student’s *t* test)

There was no significant difference in cellulase production in positive controls QM6a-A734, QM9414-A734, and PC-3-7-A734 transformants compared to the parental strains, QM6a, QM9414, and PC-3-7, respectively (Fig. 4a–e), which can be explained by the common *ace*-*734* genotype of each strain pair. However, the titers of *p*NPCase activity were significantly improved in the test QM6a-A723, QM9414-A723, and PC-3-7-A723 transformants compared to those in the parental stains (QM6a, QM9414, and PC-3-7, respectively) and in the positive controls (QM6a-A734, QM9414-A734, and PC-3-7-A734 transformants, respectively). Interestingly, the titer of *p*NPCase activity in QM9414-A723 transformants was higher than that observed in Rut-C30, one of the best-known industrial hyper-producers of cellulase. This result suggested that the truncation of ACE3 converts the moderate producer *T. reesei* QM9414 to a hyper-producer of cellulase. The PC-3-7 transformants grew poorly on lactose as the sole source of carbon and *p*NPCase activity could only be detected on Avicel. Cellulase production was significantly improved in the engineered strain PC-3-7-A723 culture, about 124% higher than the parent strain PC-3-7 production. The PC-3-7-A723 transformants showed a *p*NPCase activity of approximately 4.14 ± 0.02 U/mL in flasks, which is the highest activity that has been achieved to date. This result showed that artificially truncating 11 amino acids at the C-terminus of ACE3-734 could also enhance cellulase production in QM6a, QM9414, and PC-3-7, just simulating the classical mutagenesis caused by the missense mutation in NG14.

In addition, the transcriptional levels of *cbh1*, *xyr1*, and *ace3* were also analyzed using RT-qPCR in the QM6a group (parental strain QM6a, positive control QM6a-A734, and test QM6a-A723), in the QM9414 group (parental strain QM9414, positive control QM9414-A734, and test QM9414-A723), and in the PC-3-7 group (parental strain PC-3-7, positive control PC-3-7, and test PC-3-7) (Additional file 5: Figure S4). The transcription levels of *cbh1*, *xyr1*, and *ace3* in each pair (parental and positive control strains) are similar, probably due to their common *ace3*-*734* genotype. However, the transcription levels of *cbh1*, *xyr1*, and *ace3* in the test strains were about 20% and 100% higher than in the parental and positive control strains, respectively. The RT-qPCR analyses of *cbh1* agreed with the cellulase activity analyses (Fig. 3a–e). The slightly up-regulated transcription levels of *xyr1* and *ace3* in the test strains (Additional file 5: Figure S4) can partly explain its increased cellulase production. Hence, it can be confirmed that the truncated type ACE3-723 is a feasible strategy to enhance cellulase production in *T. reesei*.

Saccharification activity of crude cellulase from the PC-3-7-A723 transformant was first evaluated by testing its biomass saccharification ability against pretreated corn stover, using PC-3-7 and Rut-C30 as controls. The crude enzyme from the PC-3-7-A723 fermentation broth produced much more glucose than that from PC-3-7 and Rut-C30 with the same broth volume loading. After 72 h of saccharification, glucose yields of 82.48%, 60.51%, and 15.41% were achieved for cellulase from PC-3-7-A723, PC-3-7, and Rut-C30, respectively (Table 1 and Additional file 6: Figure S5). In the engineered strain PC-3-7-A723, the glucose hydrolyzed by the crude enzyme was 435% and 36% higher than in the Rut-C30 and the parent PC-3-7 strains, respectively. These results indicate that the cellulase production in PC-3-7-A723 is much better than in other strains (Table 1 and Additional file 6: Figure S5) under the same cultivating conditions, time, and material cost. Taken together, these results suggest that our engineering strategy is a useful and efficient approach to further improve cellulase production.

**Table 1:** Cellulase activities and saccharification of corn stover with same enzyme loading

Strains	Enzyme activity (U/mL)		Enzyme volume loading (mL)	Cellulase loading (FPase/g dry biomass)	Glucose (g/L)	Glucose yield (%)
	FPase	*p*NPCase				
Rut-C30	0.56 ± 0.12	0.34 ± 0.02	5	2.80	3.22 ± 0.23	15.41 ± 0.06
PC-3-7	2.05 ± 0.11	2.70 ± 0.28	5	10.25	12.64 ± 1.33	60.51 ± 0.15
PC-3-7-A723	2.95 ± 0.08	4.02 ± 0.23	5	14.75	17.23 ± 1.41	82.48 ± 0.18

### Engineered PC-3-7-A723 ferments cellulase

**Fig. 5:**
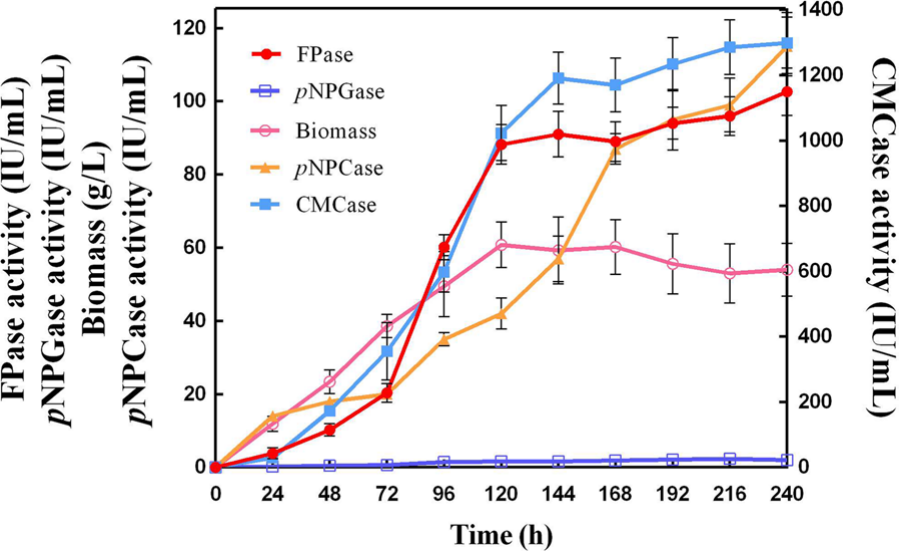
Time-course of the fed-batch culture of *T. reesei* PC-3-7-A723 for cellulase production in a 30-L fermenter. Fermentation was started with MGDS feeding. The samples were taken at regular intervals, and the supernatant was analyzed for the FPase, *p*NPCase, *p*NPGase, and CMCase activities. Mycelia were collected for biomass measurement. Values are the mean ± SD of results from three triplicate measurements

To further investigate the potential for industrial applications of engineered PC-3-7-A723, its cellulase production was analyzed in a 30**-**L fermenter. Figure 5 and Additional file 7: Table S1 illustrate the time-course of fed-batch culture using a mixture of glucose and β-disaccharides (MGDS; SUNSON^®^, Beijing, China) as the carbon source. For PC-3-7-A723, 60.8 g/L of PC-3-7-A723 biomass was obtained at 120 h, and maximum FPase activity was reached (102.63 IU/mL) at 240 h. During the fermentation process, the CMCase, *p*NPCase, *p*NPGase, and FPase activities of PC-3-7-A723 increased faster than those of PC-3-7. An increase of about 20–30% cellulase production was observed between the truncated strain PC-3-7-A723 and parental strain PC-3-7 after a 10-da cultivation (Additional file 7: Table S1). These results indicated that engineered PC-3-7-A723 is an effective strain for large-scale cellulase production and that the truncation of ACE3 is a useful strategy to increase the cellulase production.

These may be the first tests for the potential value of engineered PC-3-7-A723 as a strain for industrial-scale cellulase production.

## Discussion

Two different branches of *T. reesei* mutant strains were generated via classical mutagenesis techniques [6, 8]. Many mutations with essential roles in cellulase hyper-production still need to be identified. Several reports have identified some mutagenic events by comparative genomic screening, especially in known TFs, which provided a better understanding of the regulation in such hyper-producers of cellulase and have resulted in the construction of more efficient cellulase-producing strains through genetic engineering [5, 13, 14].

In *T. reesei* Rut-C30, three mutations have been well studied: one is a truncation in the *cre1* gene, which renders this strain carbon catabolite depressed [23]; another mutation leads to a frameshift mutation in the glycoprotein processing β-glucosidase II encoding gene [27]; the last mutation lacks a 83-kb (29 gene-encoding in the scaffold 15) region [28]. Interestingly, Rut-C30 and PC-3-7 strains, which originate from two mutation branches, possess the mutated CRE1 [6, 22]. It is suggested that the mutation in CRE1 is common in such high cellulase-secreting mutants and corresponds with a partial release from carbon catabolite repression. Mello-de-Sousa et al. [23] indicated that the truncated CRE1 contributes more to the Rut-C30 phenotype than the deletion of 29 genes encoded in the scaffold 15. But lack of the full version of CRE1 in Rut-C30 does not completely explain its hyper-production of cellulase [26]. The exact cause of enhanced cellulase production in Rut-C30 is still unclear. Our study analyzed a specific mutation of *ace3* in Rut-C30. Replacing the truncated ACE3-723 with the native ACE3-734 in *T. reesei* Rut-C30 completely blocked its cellulase hyper-production. These results suggested that the truncation of ACE3 may be much more relevant than the truncation of CRE1 for the cellulase hyper-production in *T. reesei* Rut-C30.

Rut-C30 inherits the truncated ACE3-723 from its parental strain NG14 (Fig. 1). While the M7 strain is now lost and we cannot trace the history of ACE3-723 further, the NG14 mutation branch does bear this truncation. This is the crucial cause of the cellulase hyper-production in NG14 and its descendants Rut-C30 and RL-P37 (Fig. 1c). We can imagine that the truncated ACE3-723 made NG14 competitive, thus promoting its selection as a cellulase hyper-producer 50 years ago. Our study also indicates that our approach is an effective strategy for identifying functional mutations by scanning the mutations located in TFs from the classical mutants.

In this study, we found that the transcription levels of *xyr1* and *ace3* in *T. reesei* strains with the truncated ACE3-723 were higher than those that had the native ACE3-734 (Fig. 2e, f, Additional file 4: Figure S3 and Additional file 5: Figure S4). These results showed that truncation of *ace3* leads to the overexpression of *xyr1* and *ace3*, and this subsequently enhances cellulase production in *T. reesei* [25, 29, 30]. The slight overexpression of *xyr1* and *ace3* can explain the higher cellulase expression in QM6a-A723, QM9414-A723, and PC-3-7-A723 compared to QM6a, QM9414, and PC-3-7, respectively. But how does a truncated ACE3-723 improve the transcription of *xyr1* and its own? Our previous study showed that *ace3* induces cellulase expression by interacting with XYR1, also examining which domains in ACE3 and XYR1 proteins interact with each other with a yeast 2-hybrid screening (Y2H) [26]. In this study, we found that the 11 absent amino acids in ACE3-723 are located in the protein–protein interaction domain of ACE3; this truncation may thus promote its interactions with XYR1 and indirectly enhance the transcription levels of *ace3*, *xyr1*, and cellulase-related genes, which needs further investigation. The mechanism underlying the *ace3* truncation is very impactful. But, how to explain the NG14-A734 group total loss of productivity after complementing the 11 amino acids (Fig. 3)? We hypothesize a loss of function resulting in the inability of the NG14-A734 group to sustain an increased cellulase production. We intend to construct more artificial ACE3 truncation variants to study their effect on cellulase and to identify other proteins able to interact with ACE3. This may result in an explanation to the underlying mechanism of cellulase induction by ACE3.

**Fig. 6:**
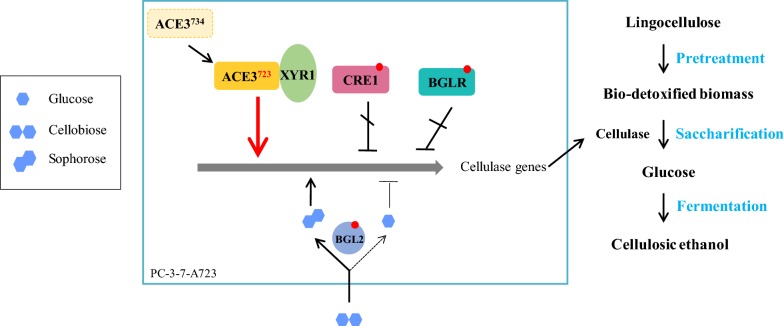
Schematic diagrams for producing cellulosic ethanol by genetic engineering of filamentous fungi *T. reesei* PC-3-7-A723. The truncated type ACE3-723 is transformed into *T. reesei* PC-3-7 to generate the engineered cellulase hyper-producer PC-3-7-A723. The truncated ACE3-723 induces cellulase expression by interacting with XYR1. PC-3-7-A723 inherits the classical mutation sites in CRE1 (releasing CCR to enhance cellulase gene expression) [22], BGLR (decreasing β-glucosidase gene expression and increasing cellulase gene expression) [24], and BGL2 (promoting the conversion of cellobiose to sophorose to induce cellulase gene expression) [13] from PC–3–7, which contribute to the improvements of cellulase. We integrated these crucial mutations in PC-3-7-A723 by rational engineering to promote the cellulase production for cellulosic ethanol

Meanwhile, two specific mutations of the PC-3-7 *bgl2* and *bglr* genes were proven to enhance cellulase expression [13, 24]. These functional mutations originated from classical random mutagenesis by scanning the overproducing mutants for improved cellulase production. We integrated these crucial mutations to generate improved cellulase production mutant strains by rational engineering in PC-3-7-A723 (Fig. 6).

Taken together, our results indicate that integrating the crucial genetic changes induced by classical mutagenesis is an effective strategy to easily and rapidly improve cellulase production. Our study also suggests that a similar ACE3-723 truncation may increase cellulase production in other *Trichoderma* species, too. Classical mutagenesis and screening have been widely employed in industry to obtain overproducing mutants for value-added products. Identifying the positive mutations and integrating them by rational genetic engineering may be useful for enhancing the production of other industrially important metabolites.

## Conclusions

In this study, we analyzed some classical mutants to plan rational engineering aimed to generate of mutant strains with improved speed and ease of production of cellulase and cellulosic ethanol. First, we identified the mutations occurring in the coding regions of TFs by scanning the genome data of hyper-productive mutants originated by classical mutagenesis. Then, we characterized these mutations to identify those crucial for the production of certain metabolites from the cells. Last, we integrated these crucial mutations to improve mutant strains by rational engineering to promote the cellular production of these metabolites.

## Methods

### Strains and growth conditions

*Escherichia coli* DH5α was used for plasmid amplification. *T. reesei* Rut-C30 (ATCC 56765), NG14 (ATCC 56767), and PC-3-7 (ATCC 66589) were purchased from ATCC (American type culture collection). *T. reesei* strain QM6a (ATCC 13631), QM9414 (ATCC 26921), and RL-P37 (NRRL 15709) were respectively purchased from DSMZ (Deutsche Sammlung von Mikroorganismen und Zellkulturen), Institute of Microbiology-Chinese Academy of Sciences, and NRRL (Agriculture Research Service Culture Collection). *E. coli* was cultured in Luria broth (LB) medium. All the strains of *T. reesei* were cultivated at 28 °C (200 rpm) in 2× Mandels’ medium (1.0 g/L yeast extract, 3 g/L peptone, 0.6 g/L urea, 2.8 g/L (NH_4_)_2_SO_4_, 4.0 g/L KH_2_PO_4_, 0.5 g/L CaCl_2_, 0.6 g/L MgSO_4_∙7H_2_O, 5 mg/L FeSO_4_∙7H_2_O, 1.6 mg/L MnSO_4_∙4H_2_O, 1.4 mg/L ZnSO_4_∙7H_2_O, and 20 mg/L CoCl_2_∙6H_2_O) in which 2% glucose, 2% lactose, or 1% (w/v) Avicel was used as carbon source [31]. In addition, all these strains were maintained on potato dextrose agar (PDA) plates at 28 °C for the generation of conidia.

### Plasmid construction, *Agrobacterium*-mediated transformation, and transformants screening

The primers used in this study are listed in Additional file 8: Table S2. LML2.1 [32] was used as a skeleton for the two plasmids, plasmid_A734_ and plasmid_A723_, to obtain two types of transformants bearing the ACE3-734 and ACE3-723 protein, respectively. The 5′- and 3′-arms of the homology double exchange for ACE3-734 and ACE3-723 sequences were constructed as described by Zhang et al. [26]. The DNA fragment of the 5′-arm of truncated ACE3-723 (approximately 900 bp) was cloned using the genome of Rut-C30 and the primer pair ace3-1/ace3-2_723_. Similarly, the fragment of the 5′-arm of native ACE3-734 (approximately 1000 bp) was cloned using the QM6a genome and the primer pair ace3-1/ace3-2_734_. The 3′-arms of these structures (about 1000 bp) were amplified from Rut-C30 or QM6a genomic DNA using the primer pair ace3-3/ace3-4. KOD-Plus-Neo (TOYOBO, Japan) was used for PCR. Next, the 5′- and 3′-arms of ACE3-723 and ACE3-734 were ligated in an orderly manner into *Pac*I/*Xba*I and *Swa*I sites of linearized LML2.1 [32] to form Plasmid_A723_ and Plasmid_A734_, respectively. The transformation experiments were performed using *Agrobacterium*-mediated transformation as described by Zhang et al. [32]. Correct intermediate transformants of A723 and A734, obtained by a homologous double exchange, were checked by diagnostic PCR [33] and quantitative PCR (qPCR; Additional file 2) [34–36] to avoid unspecific locus integration (ectopic integration events). The primer pairs ace3-CF/D70-4 and HG3.6/ace3-CR were used in diagnostic PCR (Additional file 3: Figure S2A-B), which was followed by DNA sequencing (Additional file 3: Figure S2E) to confirm the correct knock-in at the *ace3* locus in *T. reesei* genomes. The single-copy DNA fragment integration in transformed clones was verified by qPCR (Additional file 3: Figure S2C-D). The hygromycin marker gene in the transformants was excised using xylose-induced *cre* recombinase expression [32], and then the intermediate transformants were turned into the final transformants by excising the hygromycin marker gene. The final transformants were obtained and confirmed by hygromycin sensitive phenotype and with the second-round diagnostic PCR. Thus, the final A723/A734 transformants were used for further analysis.

### Cellulase production in a shake flask and fermenter culture

Cellulase production in a shake flask was conducted according to a previously described method [26], with some modification. In brief, conidia (final concentration 10^6^/mL) of *T. reesei* strains were grown at 28 °C, in 20 mL of 2× Mandels’ medium containing 2% (w/v) lactose or 1% (w/v) Avicel (PH-101, Sigma-Aldrich) as the sole carbon source. The biomass dry weights were indirectly measured by calculating the total amount of intracellular proteins [37]. The supernatant was used for cellulase assays. Mycelia were collected for RNA extraction.

The activity of the produced cellulase was measured as described in another study [38]. In brief, the *p*NPCase and *p*NPGase activities were determined against 5 mM *p*-nitrophenol-d-cellobioside (*p*NPC, Sigma-Aldrich) and *p*-nitrophenyl β-d-glucopyranoside (*p*NPG, Sigma-Aldrich) as substrates in 50 mM sodium acetate buffer at pH 5.0 at 50 °C for 30 min, respectively. The release of *p*-nitrophenol was determined by measuring absorbance at 405 nm. One unit of *p*NPCase and *p*NPGase activities was defined as 1 μmol of *p*-nitrophenol released per minute. The CMCase activities were determined by incubation in 50 mM sodium acetate buffer with 1% carboxymethylcellulose (CMC, Sigma-Aldrich), at pH 5.0, 50 °C and for 30 min. The FPase activities were determined using Whatman filter paper as the substrate with a 50 mM sodium acetate buffer at pH 5.0, 50 °C, and for 30 min. One unit of CMCase or FPase activity was defined as the amount of enzyme producing 1 μmol of reducing sugar per min.

Cellulase production in a fermenter culture was conducted according to the method described by Li et al. [39] with some modification. In brief, fermentation was carried out in the 30-L fermenter (Shanghai Bailun Bio-technology Co., Ltd.) with an initial working volume of 10 L at 28 °C for mycelial growth. Seed cultivation was performed as follows: for each strain, about 10^9^ conidia were inoculated into 1 L of 2× Mandels’ medium and 20 g/L of glucose, then cultivated by rotation (200 rpm) at 28 °C for 2 days. This culture was poured into 9 L of fresh 2× Mandels’ medium containing 10 g/L of wheat bran and 15 g/L of Avicel in a 30 L jar fermenter. A mixture of glucose and β-disaccharides (MGDS; SUNSON^®^, Beijing, China) was fed after inoculation. The feeding took place every 6 h, which maintained glucose concentration low, between 0.05 g/L and 0.30 g/L. The temperature was decreased to 25 °C after 48 h for more efficient cellulase production. The dissolved oxygen (DO) and pH were controlled as described by Li et al. [39]. The samples were taken every 24 h, and the supernatant was analyzed for the FPase, *p*NPCase, *p*NPGase, and CMCase activities. Mycelia were collected for biomass measurement.

### RNA extraction and real-time reverse-transcription polymerase chain reaction (RT-qPCR)

The methods used for RNA extraction and RT-qPCR methods are as those described by Chen et al. [33]. In brief, total RNA was extracted from cell fresh weight using a FastRNA Pro Red Kit (MPbio, Irvine, CA, USA). Synthesis of cDNA from total RNA was performed using the TransScript One-Step gDNA Removal and cDNA Synthesis SuperMix (TransGen, Shanghai, China), according to the manufacturer’s instructions. For RT-qPCR, the PerfectStart™ Green qPCR SuperMix (TransGen, Shanghai, China) was used (see Additional file 8: Table S1). The transcriptional levels of the *sar1* gene and the RNA of the parental strain were measured for reference calculation and data normalization. Primers used in RT-qPCR are listed in Additional file 8: Table S2.

### Pre-treatment and enzymatic hydrolysis of lignocellulose biomass

Dry dilute acid pretreated and bio-detoxified corn stover (containing 37.6% cellulose and 4.4% hemicellulose) was donated by Professor Jie Bao [40]. The hydrolysis efficiency of the crude cellulase was evaluated by mixing 5% (w/v) dry pretreated and bio-detoxified corn stover as a substrate and the same amount of crude enzyme (5 mL) in 50 mM phosphate buffer to a final volume of 20 mL at 50 °C and pH 5.0 for 72 h. The glucose concentration in the supernatant was determined using a glucose assay kit and glucose yield was analyzed as described by Li et al. [39]. In brief, the glucose concentration in the supernatant was measured with the GOD (glucose oxidase) method. The glucose yield was calculated as follows: Glucose yield (%) = (Glucose (g) × 0.9 × 100)/(Cellulose in substrate (g)).

### Statistical analysis

All experiments were performed with at least three independent samples with identical or similar results. The error bars indicate standard deviation (SD) of the mean of triplicates. Student’s *t* test was used to compare two samples, and Duncan’s multiple-range test was used for multiple comparisons. Within each set of experiments, *p* < 0.05 was considered significant.

## Supplementary information

**Additional file 1: Figure S1.** The DNA alignment of the native and truncated *ace3* loci from QM6a and Rut-C30, respectively. The missense mutation of C2883 to T2883 in the truncated type *ace3* eventually results in a premature termination of translation and in a truncated ACE3 protein.

**Additional file 2:** Determining copy numbers by qPCR.

**Additional file 3: Figure S2.** The verification of the transformants. (A-B) PCR amplification results of the A734 (A) and A723 (B) transformants. F were obtained using the primer pair ace3-CF/D70-4. R were obtained using the primer pair HG3.6/ace3-CR (Additional file 8: Table S1). (C) Schematic for identification of single-copy DNA integration in transformants genome. Primer pairs (ace3-T1/ace3-T2 and ace3-T3/ace3-T4 list in Additional file 8: Table S1) showed in red were used to identify the copy number of integrated genes. (D) The verification of copy numbers for A734 and A723 transformants by qPCR. The genome of QM6a is used as a reference with the single copy of native *ace3*. (E) A723 and A734 transformants were confirmed by DNA sequencing. The diagnostic PCR amplification results were sequenced. Black boxes represent the missense mutation loci in the sequence of *ace3*. Red underline represents the DNA sequence of the 11 truncated amino acids. The green dotted underlines indicate the stop codons.

**Additional file 4: Figure S3.** Effects of the native type ACE3-734 versus the truncated type ACE3-723 on the transcription of the genes encoding the major cellulase (*cbh1*) and its essential transcription factors (*ace3* and *xyr1*) in the NG14 group. Three independent experiments with three biological replicates each were performed. The *sar1* gene was used as the internal control for normalization. Values are the mean ± SD of the results from three independent experiments. Asterisks indicate a significant difference (*p < 0.05, Student’s *t* test).

**Additional file 5: Figure S4.** Construction of transformants and effects of the truncated type ACE3-723 versus the native type ACE3-734 on the transcription of genes in the QM6a group. (A) Truncation of ACE3-734 to ACE3-723 in *T. reesei* QM6a, QM9414, and PC-3-7. LML 2.1 is the erasable hygromycin selection marker in *T. reesei*. A723 transformants carry the truncated ACE3-723 as the test strains. A734 transformants bear ACE3-734 as controls. The black square denotes the *lox*P site left at the C-terminus of ACE3 after the marker was excised. The primers ace3-CF and D70-4 and HG3.6 and ace3-CR were used to verify the genotype of ACE3. (B–J) Transcription of genes encoding the major cellulase (*cbh1*) and essential transcription factors for cellulase (*ace3* and *xyr1*) were evaluated in *T. reesei* QM6a, QM9414, and PC-3-7 transformants. Three independent experiments with three biological replicates each were performed. The *sar1* gene was used as the internal control for normalization. Values are the mean ± SD of the results from three independent experiments. Asterisks indicate a significant difference (*p < 0.05, Student’s *t* test).

**Additional file 6: Figure S5.** Saccharification of corn stover by the crude enzyme from Rut-C30, PC-3-7 and PC-3-7-A723. The crude enzymes from Rut-C30, PC-3-7 and PC-3-7-A723 were mixed with 5% (w/v) corn stover and the same volumes of crude enzyme (5 mL). Values represent the mean and standard deviation of triplicate measurements.

**Additional file 7: Table S1.** Comparison of maximum cellulase activity and biomass production between PC-3-7 and PC-3-7-A723 after 240-h fed-batch fermentation in a 30-L fermenter.

**Additional file 8: Table S2.** Primers used in this study.

## Data Availability

All data generated or analyzed during this study are included in this published article [and its additional files].
