# Supplementary material for: Engineering of Trichoderma reesei for enhanced degradation of lignocellulosic biomass by truncation of the cellulase activator ACE3
Source: Biotechnol Biofuels. 2020 Apr 1;13:62. doi: 10.1186/s13068-020-01701-3 (PMC7110754; doi:10.1186/s13068-020-01701-3)
Supplement: Supplementary file 1 — Additional file 1: Figure S1. The DNA alignment of the native and truncated ace3 loci from QM6a and Rut-C30, respectively. The missense mutation of C2883 to T2883 in the truncated type ace3 eventually results in a premature termination of translation and in a truncated ACE3 protein. [file 13068_2020_1701_MOESM1_ESM.pdf]

```
native ace3 DNA     2641  GACGACCCCCCCTCTCTCTTTATATCCCTGCGGATATGTATATCATCAAGCCTCGGCACT
truncated ace3 DNA  2641  GACGACCCCCCCTCTCTCTTTATATCCCTGCGGATATGTATATCATCAAGCCTCGGCACT

native ace3 DNA     2701  TGTTGCTAATCTGTCCTGATTATGTTGTCTGGATGCTGCAGGTTGAAAAGATTCGCCTTA
truncated ace3 DNA  2701  TGTTGCTAATCTGTCCTGATTATGTTGTCTGGATGCTGCAGGTTGAAAAGATTCGCCTTA

native ace3 DNA     2761  TCGGAGCTAGTTTGCTGGCCATCATCCATCGCAACCAGGATTCACCCTTGGCTACGCGAG
truncated ace3 DNA  2761  TCGGAGCTAGTTTGCTGGCCATCATCCATCGCAACCAGGATTCACCCTTGGCTACGCGAG

native ace3 DNA     2821  CCAGGAGCGACTTTTCCGTGCTTTTGGATATTCTCACGCGGCTGGACTCGAAGGCGTCGG
truncated ace3 DNA  2821  CCAGGAGCGACTTTTCCGTGCTTTTGGATATTCTCACGCGGCTGGACTCGAAGGCGTCGG

native ace3 DNA     2881  ACCAACTGAGGAATACGTCCACTACCGTTGTTGGCTAA
truncated ace3 DNA  2881  ACTAA---------------------------------
```
