# Supplementary material for: Engineering of Trichoderma reesei for enhanced degradation of lignocellulosic biomass by truncation of the cellulase activator ACE3
Source: Biotechnol Biofuels. 2020 Apr 1;13:62. doi: 10.1186/s13068-020-01701-3 (PMC7110754; doi:10.1186/s13068-020-01701-3)
Supplement: Supplementary file 2 — Additional file 2: Determining copy numbers by qPCR. [file 13068_2020_1701_MOESM2_ESM.docx]

**1. Determining copy numbers by qPCR**

To determine the copy numbers of the DNA integration in the transformants, the fungal genomic DNAs were extracted using a Fungal DNA extraction kit (TianGen, Beijing, China). The genomic DNAs were sonicated on ice with 15 second pulses using a Bioruptor (Diagenode s.a. BELGIUM) at low power to shear DNA and then were used as the template for qPCR on the basis of the reported method [1-3]. The qPCR method was essentially the same as that described by Chen et al. [4]. The DNA fragment of a single copy gene *sar1* was used a reference. All C_T_ data were normalized to that of *sar1*, then transformants data was related to QM6a values. The primers used for the genes were listed in additional file 8: Table S2.

**References**

1. Li C, Lin F, Zhou L, et al. Cellulase hyper-production by *Trichoderma reesei* mutant SEU-7 on lactose. Biotechnology for Biofuels, 2017, 10(1):228.
2. Tisch D, Kubicek CP, Schmoll M. The phosducin-like protein PhLP1 impacts regulation of glycoside hydrolases and light response in *Trichoderma reesei*. BMC Genomics, 2011, 12(1):613.
3. Xue X, Wu Y, Qin X, et al. Revisiting overexpression of a heterologous β-glucosidase in *Trichoderma reesei*: fusion expression of the *Neosartorya fischeri* Bgl3A to *cbh1* enhances the overall as well as individual cellulase activities. Microbial Cell Factories, 2016, 15(1):122.
4. Chen Y, Wu C, Shen Y, Ma Y, Wei D, Wang W. *N*, *N*-dimethylformamide induces cellulase production in the filamentous fungus *Trichoderma reesei*. Biotechnol Biofuels. 2019;12:36.
