# Supplementary figures and images for: Engineering of Trichoderma reesei for enhanced degradation of lignocellulosic biomass by truncation of the cellulase activator ACE3
Source: Biotechnol Biofuels. 2020 Apr 1;13:62. doi: 10.1186/s13068-020-01701-3 (PMC7110754; doi:10.1186/s13068-020-01701-3)

A

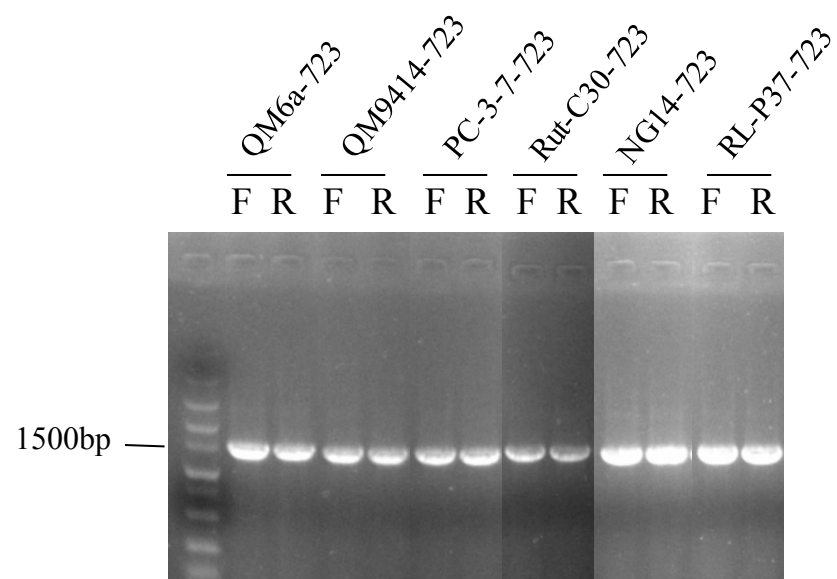

B

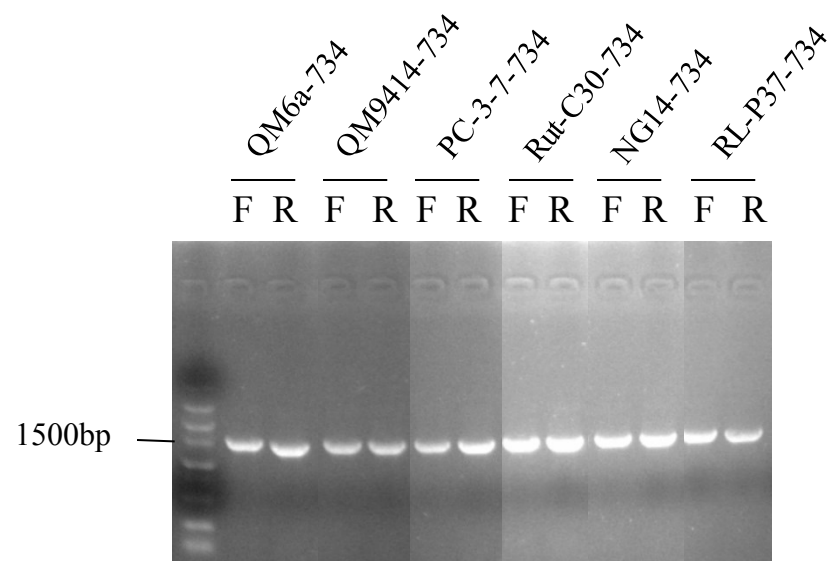

C

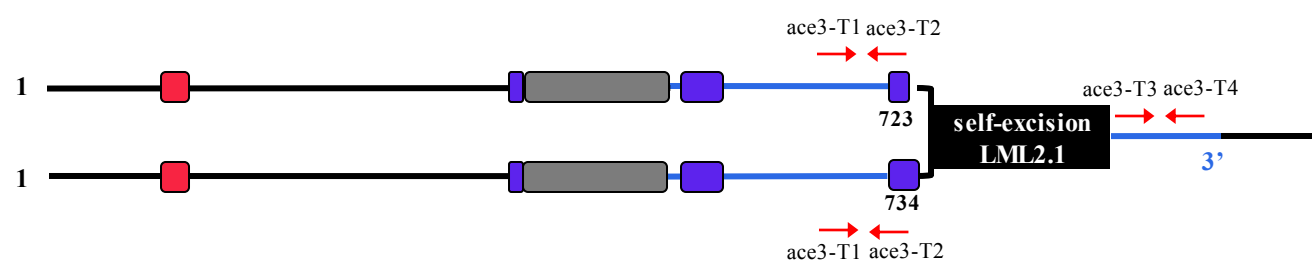

D

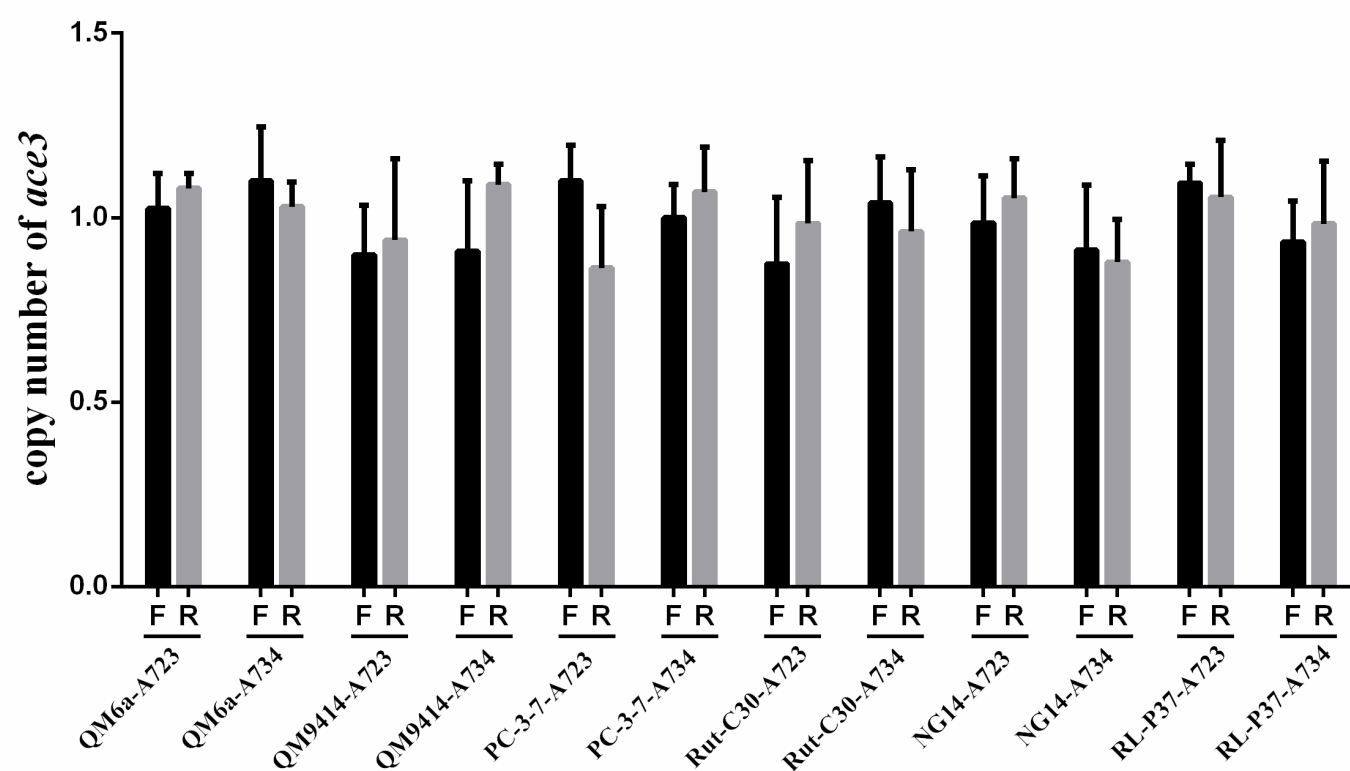

E

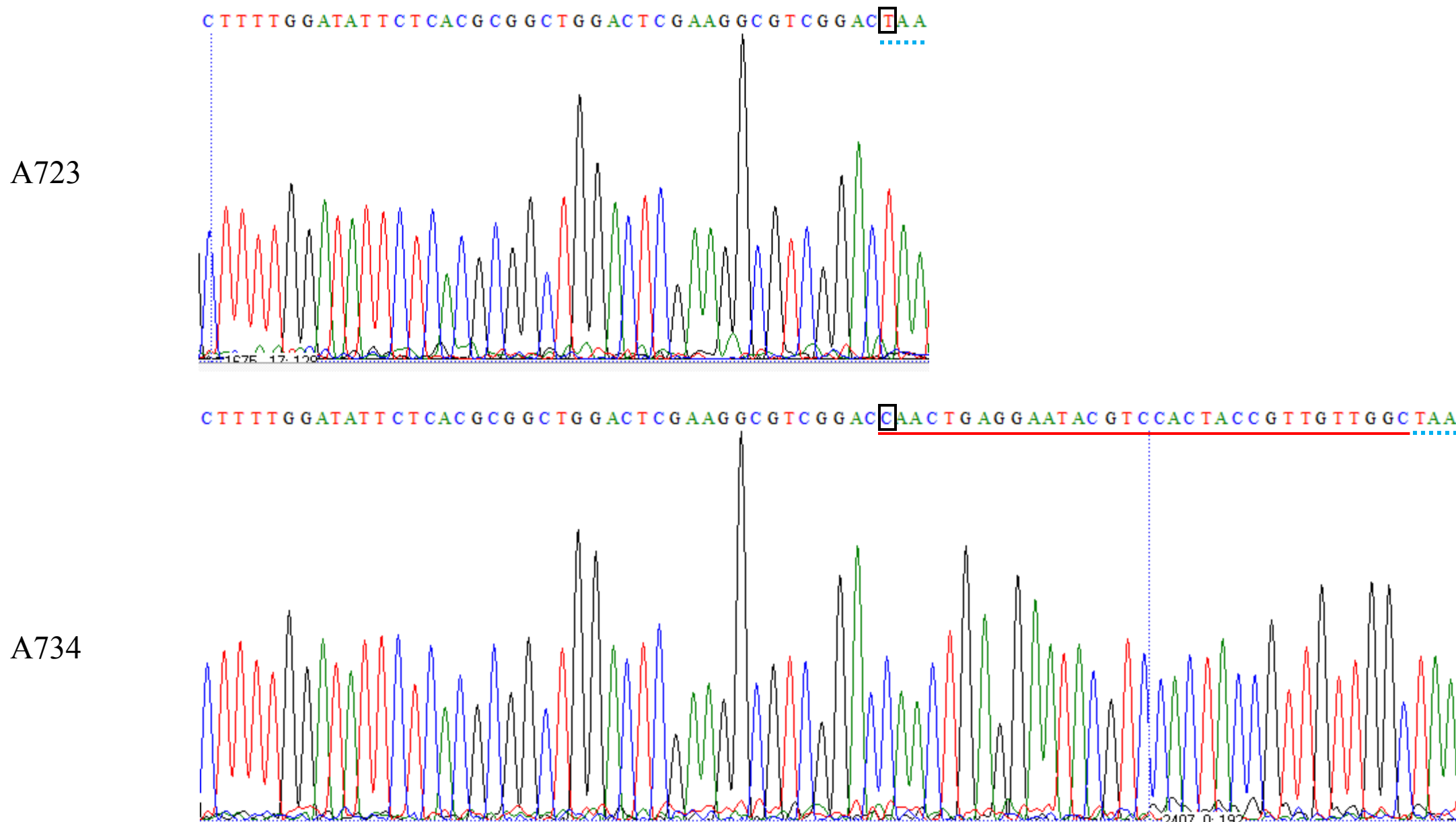

Supplement: Supplementary file 3 — Additional file 3: Figure S2. The verification of the transformants. (A-B) PCR amplification results of the A734 (A) and A723 (B) transformants. F were obtained using the primer pair ace3-CF/D70-4. R were obtained using the primer pair HG3.6/ace3-CR (Additional file 8: Table S1). (C) Schematic for identification of single-copy DNA integration in transformants genome. Primer pairs (ace3-T1/ace3-T2 and ace3-T3/ace3-T4 list in Additional file 8: Table S1) showed in red were used to identify the copy number of integrated genes. (D) The verification of copy numbers for A734 and A723 transformants by qPCR. The genome of QM6a is used as a reference with the single copy of native ace3. (E) A723 and A734 transformants were confirmed by DNA sequencing. The diagnostic PCR amplification results were sequenced. Black boxes represent the missense mutation loci in the sequence of ace3. Red underline represents the DNA sequence of the 11 truncated amino acids. The green dotted underlines indicate the stop codons. [file 13068_2020_1701_MOESM3_ESM.pdf]

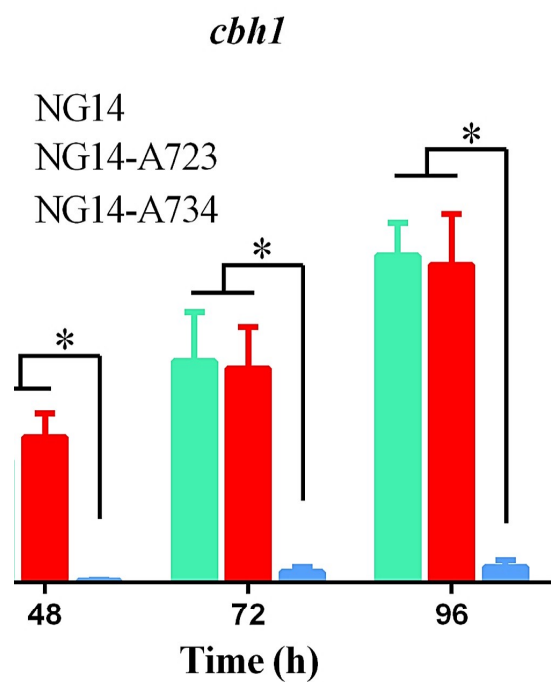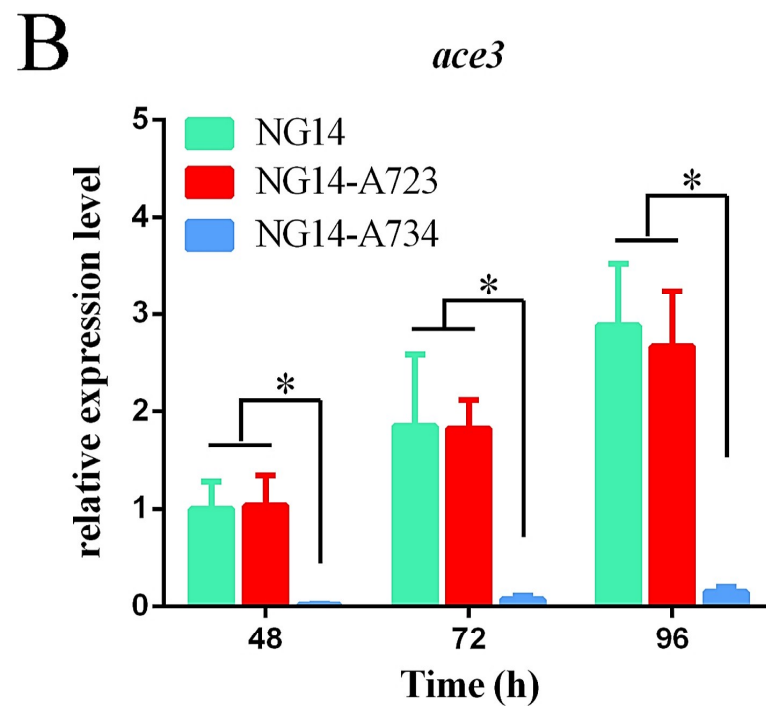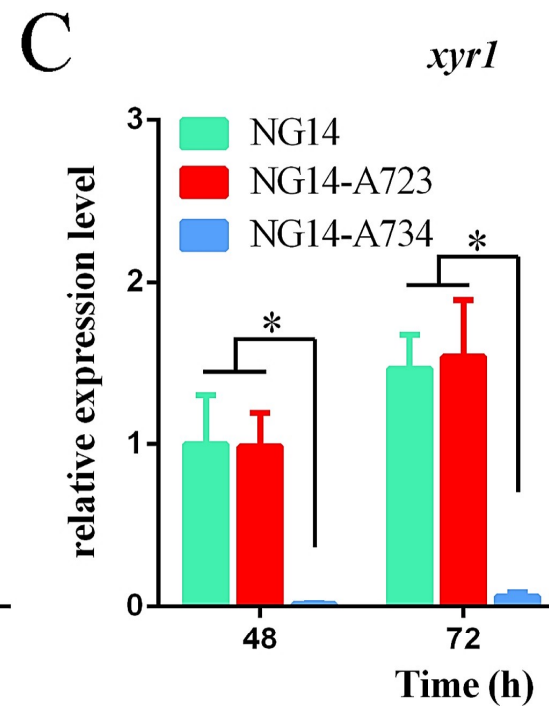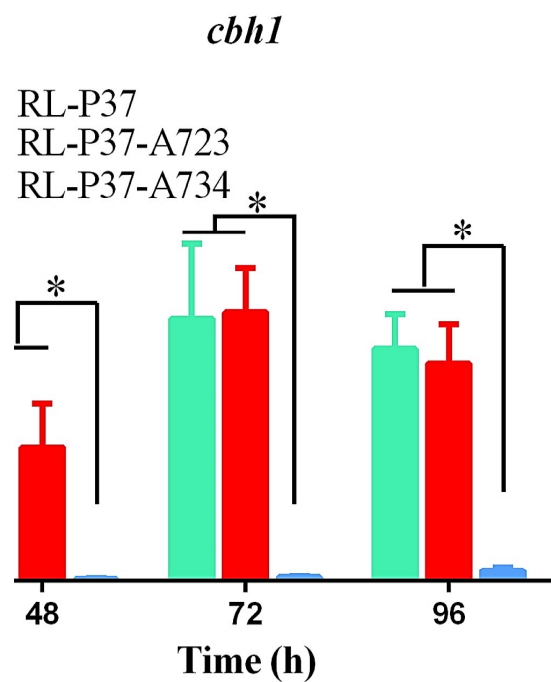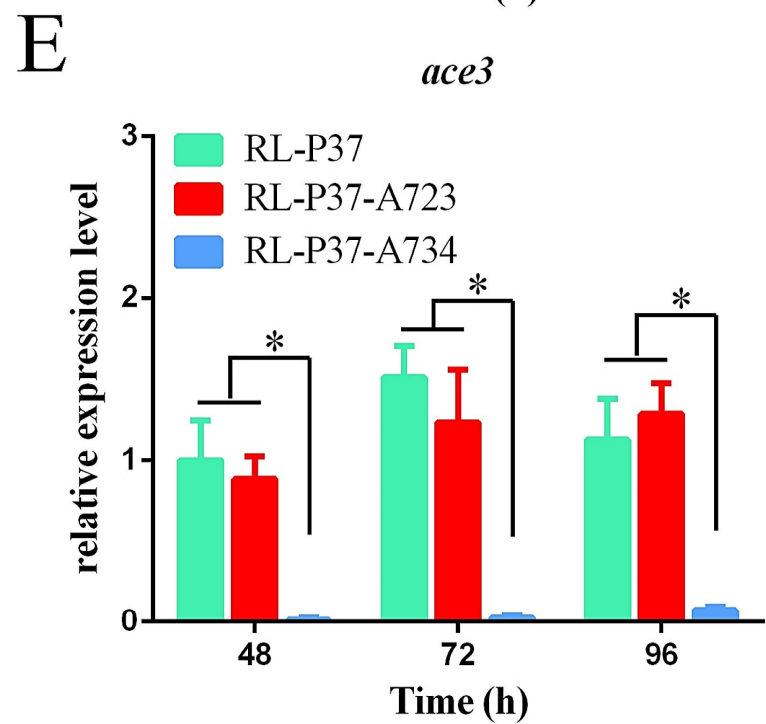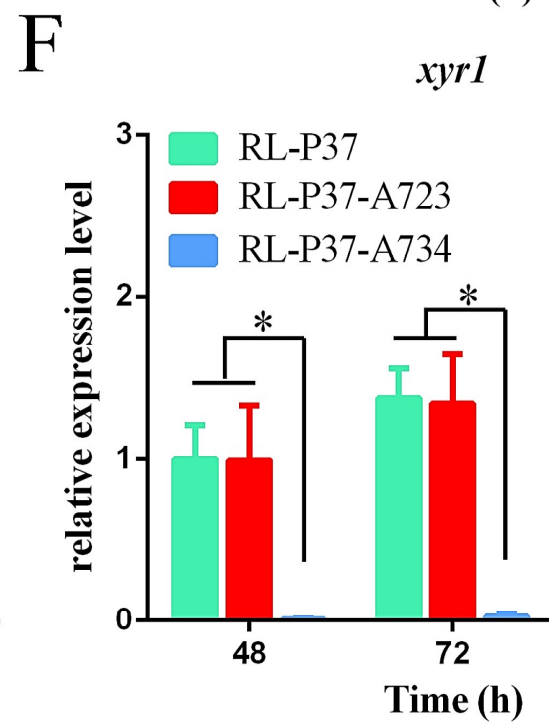

Supplement: Supplementary file 4 — Additional file 4: Figure S3. Effects of the native type ACE3-734 versus the truncated type ACE3-723 on the transcription of the genes encoding the major cellulase (cbh1) and its essential transcription factors (ace3 and xyr1) in the NG14 group. Three independent experiments with three biological replicates each were performed. The sar1 gene was used as the internal control for normalization. Values are the mean ± SD of the results from three independent experiments. Asterisks indicate a significant difference (*p < 0.05, Student’s t test). [file 13068_2020_1701_MOESM4_ESM.pdf]

A

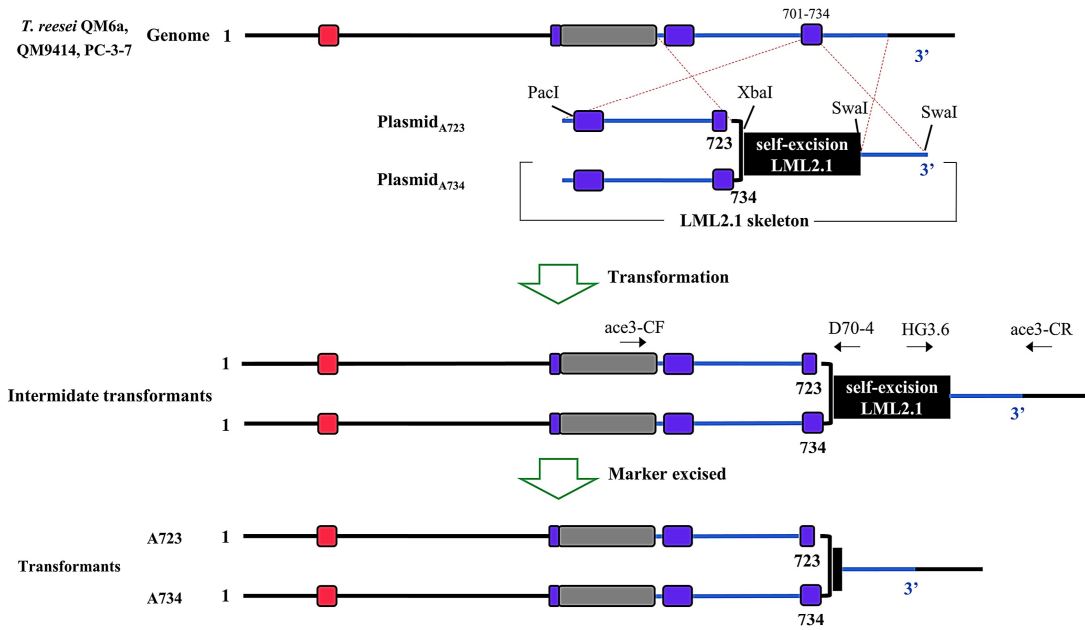

B

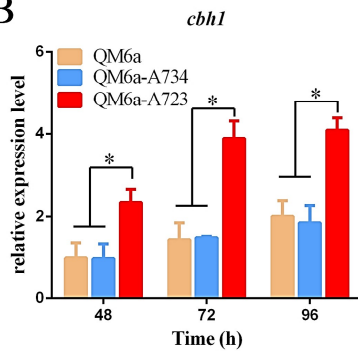

C

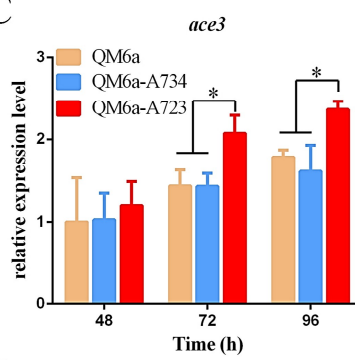

D

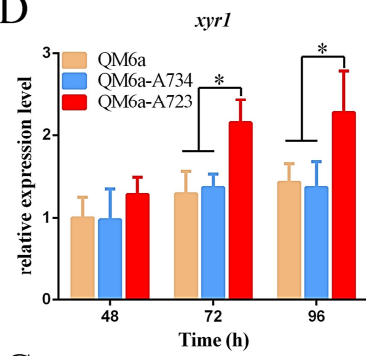

E

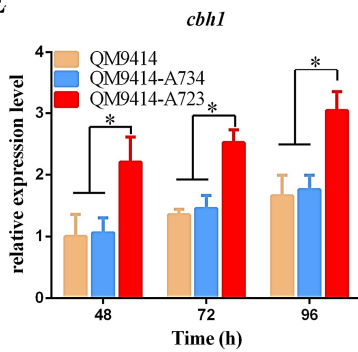

F

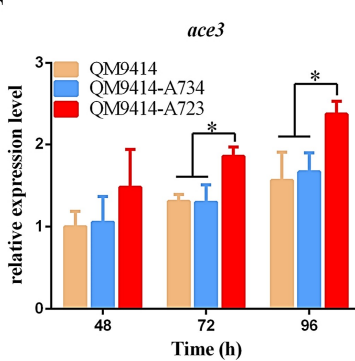

G

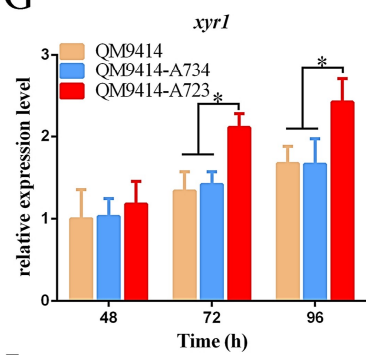

H

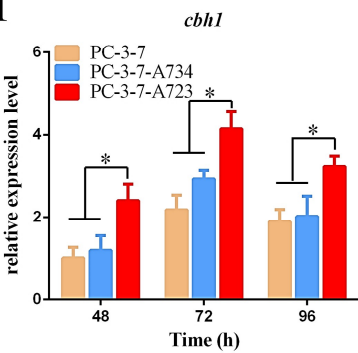

I

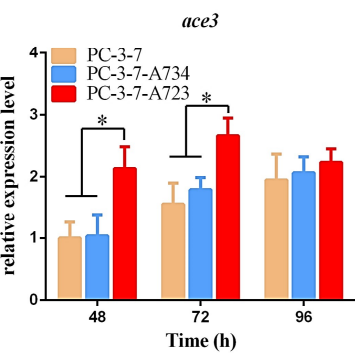

J

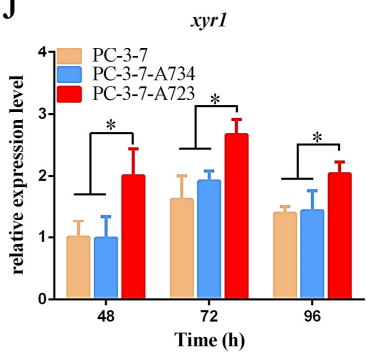

Supplement: Supplementary file 5 — Additional file 5: Figure S4. Construction of transformants and effects of the truncated type ACE3-723 versus the native type ACE3-734 on the transcription of genes in the QM6a group. (A) Truncation of ACE3-734 to ACE3-723 in T. reesei QM6a, QM9414, and PC-3-7. LML 2.1 is the erasable hygromycin selection marker in T. reesei. A723 transformants carry the truncated ACE3-723 as the test strains. A734 transformants bear ACE3-734 as controls. The black square denotes the loxP site left at the C-terminus of ACE3 after the marker was excised. The primers ace3-CF and D70-4 and HG3.6 and ace3-CR were used to verify the genotype of ACE3. (B–J) Transcription of genes encoding the major cellulase (cbh1) and essential transcription factors for cellulase (ace3 and xyr1) were evaluated in T. reesei QM6a, QM9414, and PC-3-7 transformants. Three independent experiments with three biological replicates each were performed. The sar1 gene was used as the internal control for normalization. Values are the mean ± SD of the results from three independent experiments. Asterisks indicate a significant difference (*p < 0.05, Student’s t test). [file 13068_2020_1701_MOESM5_ESM.pdf]

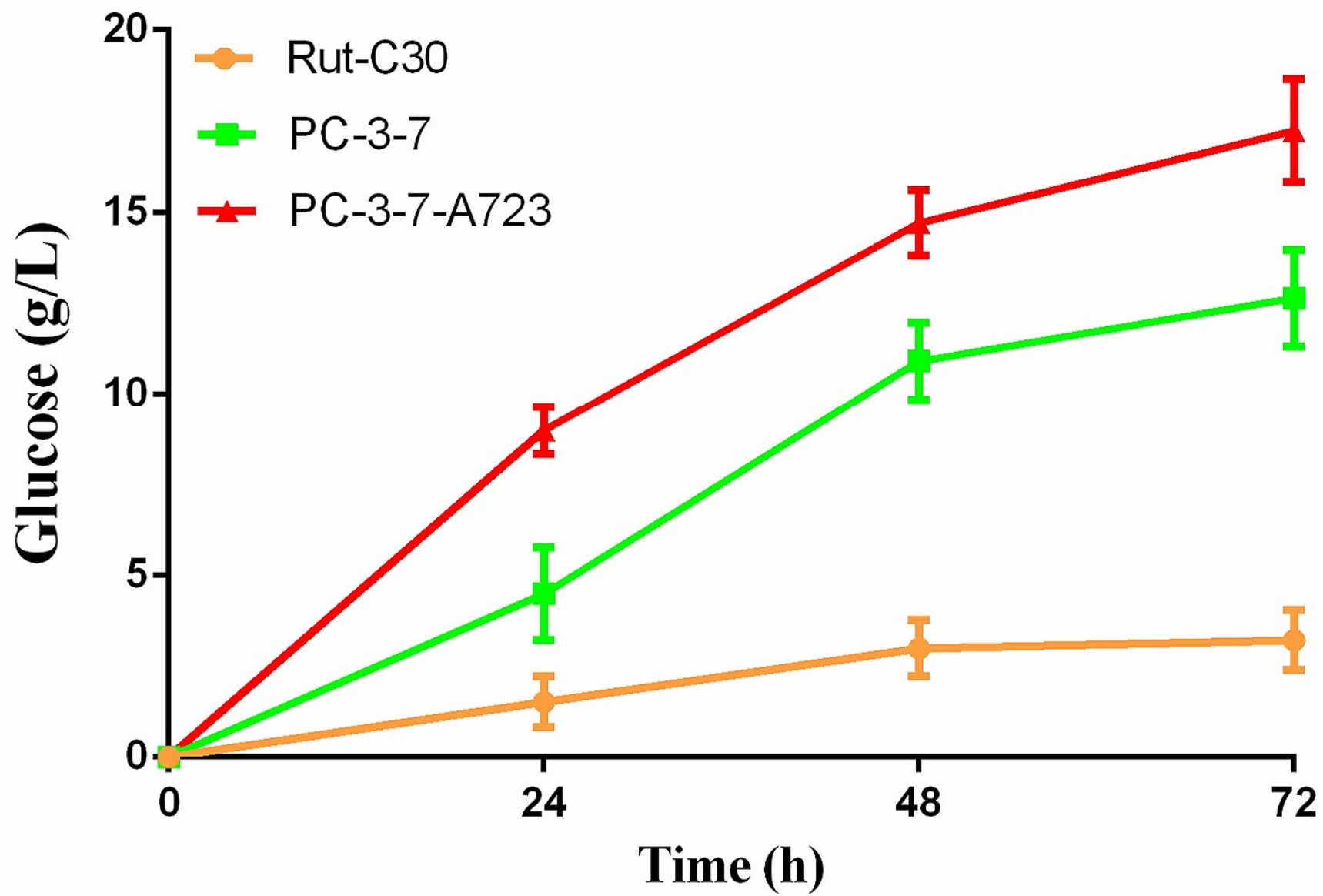

Supplement: Supplementary file 6 — Additional file 6: Figure S5. Saccharification of corn stover by the crude enzyme from Rut-C30, PC-3-7 and PC-3-7-A723. The crude enzymes from Rut-C30, PC-3-7 and PC-3-7-A723 were mixed with 5% (w/v) corn stover and the same volumes of crude enzyme (5 mL). Values represent the mean and standard deviation of triplicate measurements. [file 13068_2020_1701_MOESM6_ESM.pdf]
