# Supplementary material for: Engineering of Trichoderma reesei for enhanced degradation of lignocellulosic biomass by truncation of the cellulase activator ACE3
Source: Biotechnol Biofuels. 2020 Apr 1;13:62. doi: 10.1186/s13068-020-01701-3 (PMC7110754; doi:10.1186/s13068-020-01701-3)
Supplement: Supplementary file 7 — Additional file 7: Table S1. Comparison of maximum cellulase activity and biomass production between PC-3-7 and PC-3-7-A723 after 240-h fed-batch fermentation in a 30-L fermenter. [file 13068_2020_1701_MOESM7_ESM.docx]

**Table S1. Comparison of maximum cellulase activity and biomass production between PC-3-7 and PC-3-7-A723 after 240-h fed-batch fermentation in a 30-L fermenter.**

| Strains | FPase (U/mL) | *p*NPCase (U/mL) | CMCase (U/mL) | *p*NPGase (U/mL) | Biomass (g/L) |
| --- | --- | --- | --- | --- | --- |
| PC-3-7-A723 | 102.63±6.47 | 115.00±8.06 | 1298.70±93.25 | 2.00±0.594 | 54.00±7.26 |
| PC-3-7 | 81.08±6.21 | 93.265±7.59 | 1031.17±87.20 | 1.43±0.67 | 52.58±7.56 |

Fermentation was started with MGDS feeding. The supernatant was analyzed for the FPase, pNPCase, pNPGase, and CMCase activities. Mycelia were collected for biomass measurement. Values are the mean ± SD of the results from three independent experiments.
