# Supplementary material for: Engineering of Trichoderma reesei for enhanced degradation of lignocellulosic biomass by truncation of the cellulase activator ACE3
Source: Biotechnol Biofuels. 2020 Apr 1;13:62. doi: 10.1186/s13068-020-01701-3 (PMC7110754; doi:10.1186/s13068-020-01701-3)
Supplement: Supplementary file 8 — Additional file 8: Table S2. Primers used in this study. [file 13068_2020_1701_MOESM8_ESM.docx]

| **Table S2 Primers used in this study.**   \| Primer \| oligos Sequences (5’ to 3’) \| \| --- \| --- \| | |
| --- | --- | --- | --- |
| ace3-1 | ATTACGAATTCTTAATTAACAACCATCGCCGCTGTCACA |
| ace3-2_723_ | CATTATACGAAGTTATTCTAGATTAGTCCGACGCCTTCGAGTCC |
| ace3-2_734_ | CATTATACGAAGTTATTCTAGATTAGCCAACAACGGTAGTGGACG |
| ace3-3 | ACTAGTGAGCTCATTTGTTGTTGGCTAAATGTGTGTTGGAA |
| ace3-4 | AGTGCCAAGCTTATTTCTGTCTGTCTGTCTGCCTGTCT |
| ace3-CF | GGACGTGCTCGCATACATCTTCTC |
| D70-4 | TCGGACTTGCGGAGGATGTTGTAT |
| HG3.6 | TGCCTAGTGAATGCTCCGTAACA |
| ace3-CR  ace3-T1  ace3-T2  ace3-T3  ace3-T4  sar1-3  sar1-4 | GAAGTCTGCCCTGGTCCTCAAG  GGCCAATGGCGAGCCAAACG  GCTCGCGTAGCCAAGGGTGA  TCCCGTCATTCCGGATATTCA  CACTGCATTGCAACCCAACT  GGAGGACTCGCTGGCTTCTT  AGGATAGCAACTCGGTCGTTCT |
| Qcbh1-1 | CTCCATCTCCGAGGCTCTTACC |
| Qcbh1-2 | GCAAGTGCCGCCATATCTGTTAT |
| Qace3-1 | GCCAAGTGCGAGTACCTCAG |
| Qace3-2 | GCTGGTCGCTCTTCTTCCTC |
| Qxyr1-1 | CTTCCTCCTCCTGCTCATCG |
| Qxyr1-2 | TCGTGTGCCCTAACAATGGTC |
| Qsar1-1 | TGGATCGTCAACTGGTTCTACGA |
| Qsar1-2 | GCATGTGTAGCAACGTGGTCTTT |
